# Nitrogen Starvation Enhances the Production of Saturated and Unsaturated Fatty Acids in *Aurantiochytrium* sp. PKU#SW8 by Regulating Key Biosynthetic Genes

**DOI:** 10.3390/md20100621

**Published:** 2022-09-30

**Authors:** Xiaohong Chen, Yaodong He, Lu Liu, Xingyu Zhu, Biswarup Sen, Guangyi Wang

**Affiliations:** 1 Center of Marine Environmental Ecology, School of Environmental Science and Engineering, Tianjin University, Tianjin 300072, China; 2 Marine Traditional Chinese Medicine Research Center, Qingdao Academy of Chinese Medical Sciences, Shandong University of Traditional Chinese Medicine, Qingdao 266114, China; 3 School of Fishery, Zhejiang Ocean University, Zhoushan 316022, China; 4 Frontiers Science Center for Synthetic Biology, Key Laboratory of Systems Bioengineering (MOE), Tianjin University, Tianjin 300072, China; 5 Center for Biosafety Research and Strategy, Tianjin University, Tianjin 300072, China

**Keywords:** thraustochytrids, docosahexaenoic acid, yield, nitrogen starvation, transcriptome, mechanism

## Abstract

Nitrogen deprivation is known to improve lipid accumulation in microalgae and thraustochytrids. However, the patterns of fatty acid production and the molecular mechanisms underlying the accumulation of unsaturated and saturated fatty acids (SFAs) under nitrogen starvation remain largely unknown for thraustochytrids. In this study, batch culture experiments under nitrogen replete and nitrogen starvation conditions were performed, and the changes in the transcriptome of *Aurantiochytrium* sp. PKU#SW8 strain between these conditions were investigated. Our results showed improved yields of total fatty acids (TFAs), total unsaturated fatty acids, and total SFAs under nitrogen starvation, which suggested that nitrogen starvation favors the accumulation of both unsaturated and saturated fatty acids in PKU#SW8. However, nitrogen starvation resulted in a more than 2.36-fold increase of SFAs whereas a 1.7-fold increase of unsaturated fatty acids was observed, indicating a disproportionate increase in these groups of fatty acids. The fabD and enoyl-CoA hydratase genes were significantly upregulated under nitrogen starvation, supporting the observed increase in the yield of TFAs from 2.63 ± 0.22 g/L to 3.64 ± 0.16 g/L. Furthermore, the *pfa*B gene involved in the polyketide synthase (PKS) pathway was significantly upregulated under nitrogen starvation. This suggested that the increased expression of the *pfa*B gene under nitrogen starvation may be one of the explanations for the increased yield of docosahexaenoic acid by 1.58-fold. Overall, our study advances the current understanding of the molecular mechanisms that underlie the response of thraustochytrids to nitrogen deprivation and their fatty acid biosynthesis.

## 1. Introduction

Thraustochytrids are unicellular marine heterotrophic protists that have the unique ability to accumulate lipids up to 55% of their biomass [1,2]. Owing to their high lipid content, thraustochytrids have potential biotechnological applications, particularly in the nutraceutical and biofuel industries [3]. Certain thraustochytrids, namely the strains that belong to *Aurantiochytrium* (formerly *Schizochytrium*) genus, have shown the unique ability to accumulate large amounts of polyunsaturated fatty acids (PUFAs), especially docosahexaenoic acid (DHA) [4,5,6,7,8]. In addition to PUFAs, some studies have also suggested the high potential of thraustochytrids to accumulate saturated fatty acids (SFAs) [9,10,11,12]. Similar to other oleaginous microorganisms, the accumulation of these fatty acids in thraustochytrids is initiated when an essential nutrient limits cell division [13,14]. In recent years, there has been a growing interest in the mechanisms that underpin the connections between nutrients and biosynthetic gene expressions in thraustochytrids [15,16,17,18].

The unique fatty acids that are produced by different species or strains of thraustochytrids depend on the culture conditions. For example, nitrogen source plays a crucial role in the regulation of cellular growth and lipid biosynthesis in thraustochytrids [7,19,20]. Several studies have shown that a high carbon-to-nitrogen ratio is advantageous for lipid accumulation in thraustochytrids grown in complex media containing yeast extract [21,22,23] or corn steep liquor [24]. The specific productivity (q) of total fatty acids (TFAs) produced by *Aurantiochytrium* sp. T66 under nitrogen starvation (q = 10 mg/g-h at 112 h) was two-fold that under nitrogen in excess (q = 5 mg/g-h at 140 h) [15]. A previous study reported that nitrogen starvation induces lipid and DHA biosynthesis but limits cellular growth in *Aurantiochytrium* sp. [25]. Furthermore, nitrogen limitation combined with O_2_ limitation can raise the lipid content to 63% (*w*/*w*) of dry cell weight in *Aurantiochytrium* sp. T66 [19]. How nitrogen limitation regulates the fatty acids biosynthesis in thraustochytrids at a molecular level remains less studied [13,20].

Two distinct enzyme complexes, namely the fatty acid synthase (FAS) and PUFA synthase are known to be involved in the de novo biosynthesis of DHA in thraustochytrids [26,27,28]. However, these pathways lack the full complement of genes for standalone DHA synthesis and their relative contribution to the synthesis of DHA remains unclear [29]. Previous studies reported that genes involved in assembling the triacylglycerols (TAGs) and synthesis of the lipid body membrane were strongly upregulated after nitrogen depletion, whereas the FAS gene was not, or only slightly, upregulated in *Schizochytrium* spp. [18,30], *Yarrowia lipolytica* [31], and *Mortierella alpina* [32]. There was no upregulation of genes involved in the generation of precursors (NADPH and acetyl-CoA) in the *Schizochytrium* spp. and *Y*. *lipolytica*. However, a strong upregulation of the NADPH-generating enzymes of the pentose phosphate pathway was observed during lipid accumulation in *Mortierella alpina* [32]. Other studies reported that isocitrate dehydrogenase (ICDH) and malic enzyme (ME) provide the NADPH for fatty acids biosynthesis upon nitrogen exhaustion and facilitate lipid and DHA accumulation in *Schizochytrium* sp. ATCC 20888 [17]. Overall, the previous studies on thraustochytrids suggest that one or more genes of lipid biosynthesis pathways may show differential regulation upon nitrogen deprivation. 

In this study, a laboratory strain, *Aurantiochytrium* sp. PKU#SW8 was used to investigate the differential regulation of fatty acid biosynthesis between nitrogen replete and nitrogen starvation conditions. The molecular mechanisms of fatty acid biosynthesis under these two conditions were revealed through the analysis of global transcriptomes. The ultimate goal is to understand the patterns of fatty acid production and the molecular mechanisms underlying the differential accumulation of fatty acids in thraustochytrids under nitrogen starvation.

## 2. Results and Discussion

### 2.1. Effect of Nitrogen Concentration on Biomass and DHA Production 

**Figure 1:**
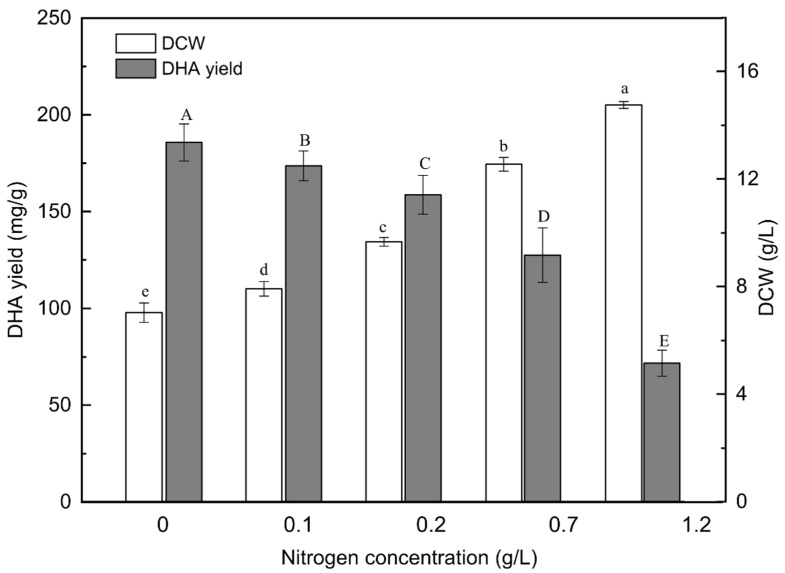
Effects of nitrogen concentration on the DCW and DHA yield of *Aurantiochytrium* sp. PKU#SW8 culture. Each bar represents the mean ± SD of triplicate experiments. Data are provided for samples taken at 96 h of batch fermentation. The carbon source was glucose and its concentration in the medium was 20 g/L. The incubation time of the culture was 96 h. One-way analysis of variance (ANOVA) was used to determine the statistically significant differences between the means. The significant differences (alpha = 0.05) of DCW and DHA yield are indicated by different lowercase and uppercase letters, respectively.

To understand the effect of nitrogen concentration on the biomass and DHA production of PKU#SW8, different concentrations (0–1.2 g/L) of monosodium glutamate (MSG) in the culture medium were tested. MSG has been reported to be one of the optimal nitrogen sources for high yields of DHA (g per g biomass) produced by thraustochytrids [7,17,33]. A defined nitrogen source such as MSG can generally be easily utilized by microorganisms for rapid cell growth and the glutamate can be converted into other nitrogen-containing compounds [34]. In this study, with increasing concentrations of nitrogen, the biomass increased almost linearly (R^2^ = 0.96), reaching the highest concentration (14.75 ± 0.13 g/L) at the nitrogen concentration of 1.2 g/L (Figure 1). A similar increasing trend of biomass production was reported when *Aurantiochytrium* sp. was cultivated on MSG as the nitrogen source [35]. 

The highest (1.60 ± 0.18 g/L) and lowest (1.07 ± 0.09 g/L) DHA concentrations were achieved at a nitrogen concentration of 0.7 g/L and 1.2 g/L, respectively. Whereas, the highest DHA yield (185.6 mg DHA per g of biomass) was at the nitrogen concentration of 0 g/L, i.e., when the cells were nitrogen starved (Figure 1). Similar DHA yields were reported in previous studies where peptone was used as the nitrogen source for the cultivation of PKU#SW8 strain [7,20]. Similarly, nitrogen starvation of *Aurantiochytrium* sp. strain T66 resulted in the production of DHA up to 29% of TFAs [19]. These results suggest that nitrogen starvation can be a useful strategy for promoting the DHA yields of certain *Aurantiochytrium* strains.

### 2.2. Patterns of Fatty Acids under Nitrogen Replete and Starvation Conditions

**Figure 2:**
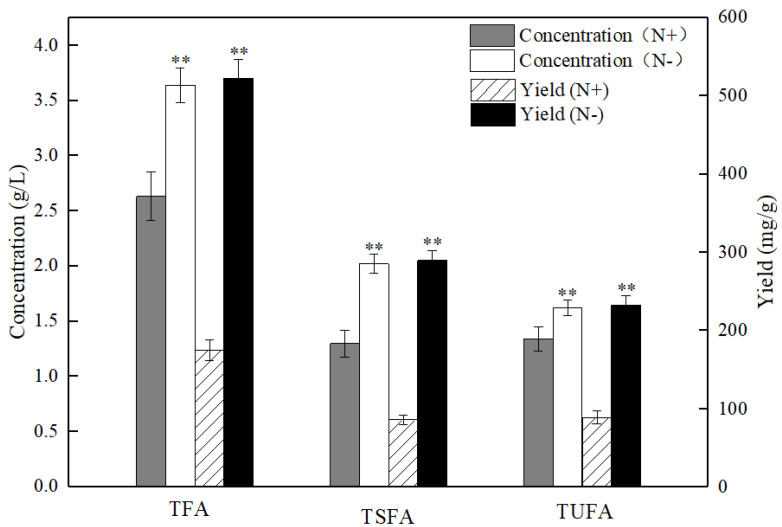
Comparison of the concentrations and yields of total fatty acids (TFA), total unsaturated fatty acids (TUFA), and total saturated fatty acids (TSFA) in *Aurantiochytrium* sp. PKU#SW8 cells grown between nitrogen replete (N+) and nitrogen starvation (N-) conditions. Data are provided for samples taken at 96 h of batch fermentation. The carbon source was glucose and its initial concentration was 20 g/L. The yield represents the fatty acid content of the biomass. Each bar represents the mean ± SD of triplicate experiments. One-way analysis of variance (ANOVA) was used to determine the statistically significant differences between the means. ** indicate the significant differences between the group means of TFA, TSFA, or TUFA at alpha = 0.01.

The accumulation of various intracellular fatty acids in the PKU#SW8 strain under nitrogen starvation was compared with that of under nitrogen replete conditions. The concentrations of nitrogen for the nitrogen starvation and nitrogen replete conditions were set to 0 g/L and 1.2 g/L, which were determined based on the results obtained in this study (see Section 2.1). The comparative results showed that the PKU #SW8 strain accumulated significantly (*p* < 0.01) higher concentrations and yields of TFAs under the nitrogen starvation condition (Figure 2). The yields of TFAs under nitrogen starvation and nitrogen replete conditions were 522.2 ± 24.4 mg/g and 175.1 ± 13.6 mg/g, respectively. Thus, nitrogen starvation resulted in an almost two-fold increase in TFAs in PKU#SW8. The increase in TFAs under nitrogen-limited culture conditions has also been reported in previous studies [21,22]. When the nitrogen sources provided in the medium are used up by the cells, the division and proliferation rate of thraustochytrids decreases or even stops. However, glucose can still be transported into the cells, and the excess glucose transported into the cells can be used to synthesize fatty acids for storage [27]. 

**Figure 3:**
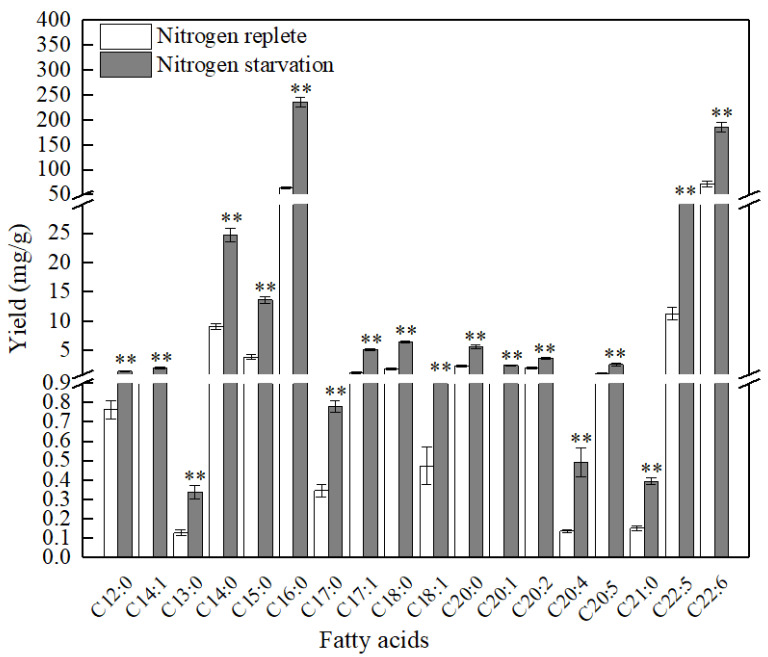
Comparison of fatty acid production by *Aurantiochytrium* sp. PKU#SW8 between nitrogen replete and nitrogen starvation conditions. Data are provided for samples taken at 96 h of batch fermentation. Each bar represents the mean ± SD of triplicate experiments. For each fatty acid, Students *t*-test was used to determine the statistically significant differences between the means. ** indicate the significant differences at alpha = 0.01.

Interestingly, nitrogen starvation favored the accumulation of both SFAs and unsaturated fatty acids in PKU#SW8 (Figure 3). The yields of these two groups of fatty acids under nitrogen starvation were significantly (*p* < 0.01) higher than those under nitrogen replete conditions. However, nitrogen starvation resulted in more than a 2.36-fold increase of SFAs, whereas a 1.7-fold increase of unsaturated fatty acids was observed. Particularly, more than a two-fold increase in C15:0 (pentadecanoic acid), C16:0 (palmitic acid), C18:0 (stearic acid), and more than a three-fold increase in C17:1 (heptadecenoic acid) were observed under nitrogen starvation. In a previous study, *Aurantiochytrium* sp. T66 also showed a significant increase in the production of SFAs under nitrogen limitation conditions, whereas the increase in unsaturated fatty acids production was small [36]. These results suggest that nitrogen deprivation of certain *Aurantiochytrium* strains can cause a disproportionate accumulation of intracellular fatty acids. It is therefore likely that nitrogen starvation differentially regulates the accumulation of fatty acids in thraustochytrids via different biochemical pathways. 

### 2.3. Relative Changes in PKU#SW8 Transcriptome under Nitrogen Starvation

The results of this study indicated that nitrogen starvation can enhance both saturated and unsaturated fatty acids in PKU#SW8 strain. To further understand the molecular mechanisms that underline the increased accumulation of these fatty acids, transcriptomic analysis of fatty acid biosynthesis under nitrogen starvation relative to the nitrogen replete condition was carried out. After removing the low-quality reads and rRNA sequences, we obtained 24,491,255 (nitrogen replete) and 22,574,182 (nitrogen starvation) clean reads with a total of 42.18 Gigabase (Gb) of clean reads. The clean data of all samples reached 6.41Gb and the percentage of Q30 base was above 93.97%. Clean reads of each sample were sequentially aligned with the reference genome. The alignment efficiency ranged from 97.43% to 97.79% (Table S1). Based on the alignment results, variable splicing prediction, gene structure optimization, and new gene discovery were conducted (Figures S1–S3); a total of 303 new genes were discovered (Table S2), 22 of which were functional annotations (Table S3). 

**Figure 4:**
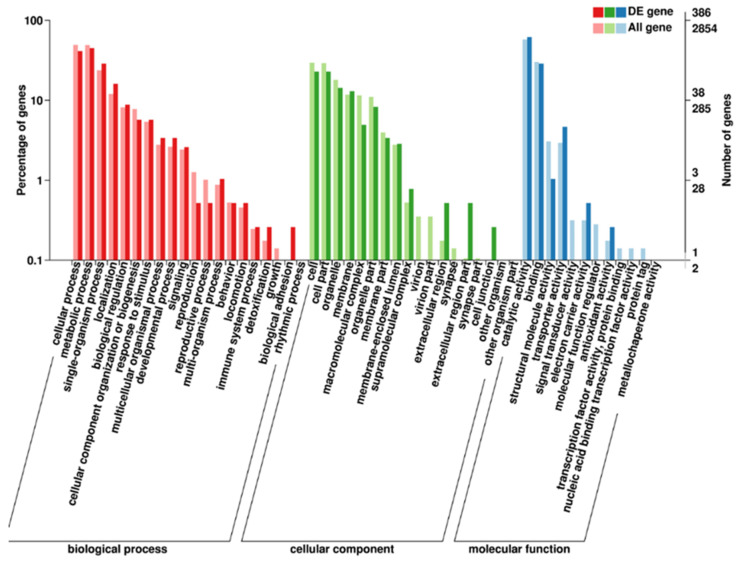
Gene Ontology classification (*p* ≤ 0.001) of DEGs under nitrogen starvation. The strain *Aurantiochytrium* sp. PKU#SW8 was used in RNA-seq experiments. The strain was cultivated on glucose (20 g/L) as the carbon source and MSG (0 g/L and 1.2 g/L) as the nitrogen source for 96 h.

Furthermore, we identified 2116 DEGs, comprising 887 upregulated and 1229 downregulated, when q-value ≤ 0.05 and log2 fold change ≥1 were applied as the cutoff values. A majority of these DEGs were significantly enriched in the biological process, followed by cellular components and molecular function (Figure 4). To validate the RNA-seq results, certain DEGs were chosen for verification by qPCR experiments. The qPCR results showed that the expression levels of those genes were mostly consistent with the RNA-seq results (Table 1), indicating the reliability of the RNA-seq data. The DEGs related to carbohydrate and lipid metabolism, including glycolysis, gluconeogenesis, TCA cycle, fatty acids biosynthesis, fatty acids metabolism, fatty acids biosynthesis, biosynthesis of unsaturated fatty acids, fatty acids elongation pathway, fatty acids degradation, fatty acids synthase (FAS), and polyketide synthase (PKS) pathways were further analyzed.

**Figure 5:**
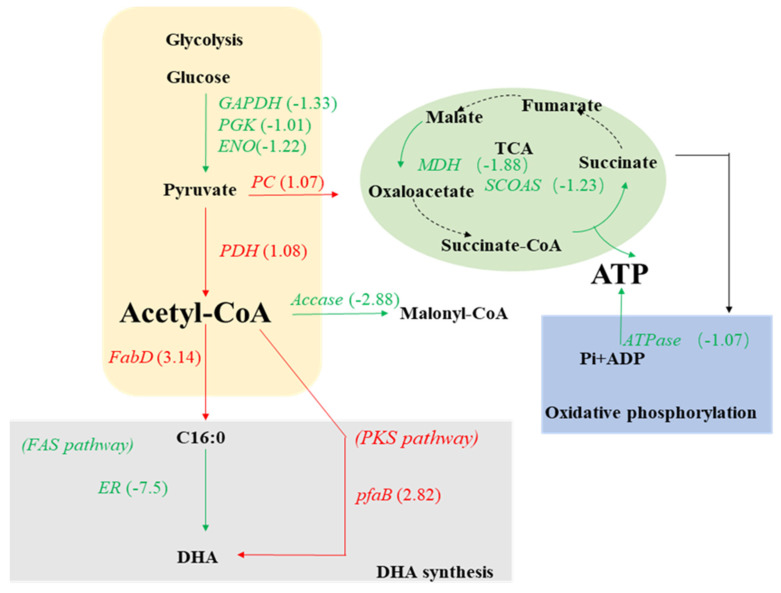
Transcriptional regulation of the central carbon metabolic pathways in *Aurantiochytrium* sp. PKU#SW8 under nitrogen starvation.

Under nitrogen starvation, the glyceraldehyde 3-phosphate dehydrogenase (GAPDH), phosphoglycerate kinase (PGK), and enolase (ENO) encoding genes were downregulated (Figure 5), which may have resulted in the downregulation of the glycolytic pathway, ultimately leading to the decrease in glucose to pyruvate conversion. This is consistent with a previous study in which nitrogen limitation weakened the glucose metabolism in *Phaeodactylum tricornutum* and made the carbon flow more into lipid biosynthesis via the pentose phosphate pathway [37]. Furthermore, the gene encoding pyruvate dehydrogenase (PDH) enzyme which catalyzes pyruvate to acetyl-COA was significantly upregulated in the PKU#SW8 strain (Figure 5), which might have resulted in the increased flux of acetyl-COA to fatty acids biosynthetic pathways.

The malate dehydrogenase (MDH) and succinyl-CoA synthetase (SCOAS) encoding genes involved in the tricarboxylic acid (TCA) cycle were downregulated (Figure 5), which indicated that nitrogen starvation can also affect the TCA cycle and biosynthetic precursors. Particularly, the downregulation of SCOAS, which catalyzes the reversible reaction of succinyl-CoA to succinate, possibly indicated that the resulting increased pool of succinyl-CoA can act as a precursor for the biosynthesis of certain amino acids. As cells are deprived of nitrogen, they may utilize those synthesized amino acids for growth. 

Furthermore, the relative expression levels of the genes encoding ICDH and ME upon nitrogen starvation did not change significantly. This result indicated that NADPH required for fatty acids biosynthesis in PKU#SW8 strain under nitrogen starvation was not provided mainly by the TCA cycle. ICDH and ME are two important enzymes in the TCA cycle and previous studies on their activities in *Schizochytrium* sp. ATCC 20888 in response to ammonia exhaustion in the culture medium showed that nitrogen exhaustion decreases the activities of these two enzymes [17].

The expression of [acyl carrier protein] s-malonyl transferase gene (fabD) was significantly upregulated (Figure 5), which has been previously reported to increase the production of fatty acids in *E. coli* [38]. This was consistent with the fermentation data that showed an increase in the production of TFAs from 2.63 ± 0.22 g/L to 3.64 ± 0.16 g/L when PKU#SW8 was cultured under nitrogen starvation. Moreover, the enoyl-CoA hydratase gene essential to metabolizing fatty acids in beta-oxidation was significantly downregulated by 1.99-fold under nitrogen starvation (Table 1), supporting the increased accumulation of TFAs. Furthermore, the expression of the polyketide synthase subunit B (*pfa*B) gene involved in the polyketide synthase (PKS) pathway was significantly upregulated, perhaps suggesting an elevated activity of the PKS pathway. In previous research [20], although there was a significantly upregulated expression of the *pfa*B gene under nitrogen starvation, the DHA concentration did not increase concomitantly. Interestingly, in this study, there was a 1.58-fold increase in DHA yield (mg/g biomass) under nitrogen starvation. On the other hand, a previous study on *Aurantiochytrium* sp. T66 showed significant upregulation of FAS under nitrogen-deficient conditions, but only marginal upregulation of the PUFA-synthase genes [15]. 

Taken together, these results raise the possibility of multiple mechanisms for the increased levels of unsaturated fatty acids when thraustochytrids are cultivated in a nitrogen-deprived medium. 

## 3. Materials and Methods

### 3.1. Strain and Culture Media

A previously isolated thraustochytrid strain, *Aurantiochytrium* sp. PKU#SW8 (CGMCC No. 20069), was used in this study [39]. The purified strain was maintained on 2% modified Vishniac’s (MV) agar plates prepared with 100% artificial seawater (ASW, 33 g/L sea salt) at 28 °C [40]. The strain was subcultured every four weeks on 2% MV agar plates. The seed culture was prepared by inoculating a single colony from the 2% MV agar plate into an M4 medium (glucose, 20 g/L; peptone, 1.5 g/L; yeast extract, 1.0 g/L; KH_2_PO_4_, 0.25 g/L; 100% ASW, and pH 7.0) [41] and incubating at 28 °C on an orbital shaker at 170 rpm for 36 h.

### 3.2. Batch Culture Experiments

To study the effects of different nitrogen concentrations on the DHA yield, batch culture experiments were carried out in two sequential stages. In the first stage, the seed culture (5% *v*/*v*) was transferred to a 100 mL shake flask containing 50 mL fresh M4 medium and incubated at 28 °C on an orbital shaker at 170 rpm for 72 h. In the second stage, the cells harvested at 72 h of fermentation in the first stage were centrifuged (4000 rpm, 4 °C, 10 min) and washed twice with sterile ddH_2_O. The resulting cell pellet was transferred to a 100 mL shake flask containing 50 mL fresh M4 medium (glucose, 20 g/L; KH_2_PO_4_, 0.25 g/L; nitrogen, 0–1.2 g/L; 100% ASW, and pH 7). The nitrogen source in the M4 medium was replaced with monosodium glutamate (MSG). The culture was cultivated at 28 °C on an orbital shaker at 170 rpm for seven days. All experiments were conducted in triplicate.

### 3.3. Quantification of Biomass and Fatty Acids

*Aurantiochytrium* sp. PKU #SW8 cells were collected by centrifugation (4000 rpm, 4 °C, 10 min) and washed twice with distilled water followed by lyophilization for 48 h. The dry cell weight was determined by the gravimetric method. Fatty acid methyl esters were prepared using the direct transesterification method [42] and analyzed following the procedures described in our previous study [43].

### 3.4. Transcriptome Analysis

Samples for the transcriptome analyses of nitrogen-replete and nitrogen-starved cells were collected from the cultures grown on 0 g/L and 1.2 g/L of MSG, respectively. The samples were taken at 96 h of fermentation, which was based on the preliminary experimental data that suggested maximum concentration and yield of DHA production at 96 h (data not shown). Experiments were performed in triplicates for each condition. The total RNA was isolated from a fresh culture (5 mL) of *Aurantiochytrium* sp. PKU#SW8. The culture broth was centrifuged at 12,000 rpm for 5 min at 4 °C and the resulting cell pellet was used for total RNA isolation using the Trizol extraction method. The isolated total RNA was quantified on NanoDrop 2000c (Thermo Fisher Scientific, Waltham, MA, USA). Genomic DNA was removed with gDNase and cDNA was synthesized using random hexamers with a FastKing RT kit (Tiangen, Beijing, China). The synthesized cDNA was used as the template for qPCR experiments.

mRNA was purified from total RNA with poly-T oligo-attached magnetic beads. The first strand of cDNA was synthesized using random hexamer primers after fragmentation and the second strand was synthesized using RNase H and DNA polymerase I. The overhangs were converted into blunt ends, the 3′ ends of the cDNA fragments were adenylated, and the adaptors were ligated to the fragments. The cDNA fragments with a specific length were separated by agarose gel electrophoresis and purified. The size-selected cDNA fragments were PCR amplified to prepare the library, which was then sequenced on an Illumina HiSeq 2500 platform by Biomarker Technologies (Beijing, China).

Raw sequencing reads were filtered by removing reads with adapter and those containing more than 10% unknown bases or having low quality. Clean reads were then mapped to Ribosomal Database Project using Bowtie 2 [44] and the reads that belonged to rRNA were removed. The remaining clean reads were mapped to the reference genome (PKU#SW8 genome) by TopHat2 [45]. The transcriptome was assembled using the Cuffmerge script in Cufflinks [46]. Differential expression analysis was performed with the *edgeR* package [47]. The gene expression level was normalized by the FPKM (fragments per kilobases of transcript per million mapped reads) method. FDR (false discovery rate) was used to determine the p-value threshold in multiple tests. The genes with FDR ≤ 0.05 and the absolute value of log2 fold change ≥1 were regarded as differentially expressed genes (DEGs). All downstream sequencing analyses were performed by Biomarker Technologies (Beijing, China). 

### 3.5. Validation of RNA-Seq Experiments by qPCR 

Quantitative PCR assays were performed on the CFX Connect Real-Time PCR system (Bio-Rad, USA) using the CharmQ SYBR qPCR master mix (Vazyme, China). The gene-specific primer sequences (Table S1) were designed by Primer Premier 6.0 (PREMIER Biosoft International, Palo Alto, CA, USA). PCR amplification was carried out in a 10 μL reaction volume containing 5 μL of qPCR master mix, 0.25 μL of each primer (10 μM), 1 μL cDNA, and 3.5 μL nuclease-free water. The PCR program was set to 95 °C for 3 min followed by 40 cycles of amplification steps at 95 °C for 20 s, 55 °C for 20 s, and 72 °C for 30 s. The expression level of each target gene relative to β-actin was calculated following the 2^−ΔΔCt^ method [48]. The amplification efficiencies for target and reference genes ranged between 101.48% and 106.16%. Melt curve analysis showed a single peak for each of these genes. All assays were conducted in triplicate.

### 3.6. Statistical Analysis

Data are expressed as mean ± standard deviation (SD). ANOVA and Students T-test were used to determine the statistically significant differences between the means of three or more groups and between two groups, respectively, using IBM SPSS Statistics software. Prior to performing ANOVA, the homogeneity of variance was tested using Levene’s test in IBM SPSS Statistics software.

## 4. Conclusions

This study provides the first evidence for the differential regulation of key fatty acid biosynthetic genes in the transcriptome of *Aurantiochytrium* sp. PKU#SW8 under nitrogen starvation. The fermentation data suggested a significant increase in the accumulation of fatty acids, including SFA and unsaturated fatty acids. RNA-seq results revealed the regulation of key genes involved in the biosynthesis of these fatty acids. Particularly, the fabD and *pfa*B genes were significantly upregulated under nitrogen starvation, which was one of the possible explanations for the increased yield of TFAs. In addition, the enoyl-CoA hydratase gene involved in the beta-oxidation of fatty acids was significantly downregulated, which further supported the observed increase in TFA yield. Interestingly, this study showed the increased expression of the *pfa*B gene concomitant with the observed increase in DHA yield in thraustochytrids for the first time. However, more research on molecular mechanisms needs to be undertaken before the association between nitrogen starvation and disproportionate accumulation of intracellular fatty acids in thraustochytrids is more clearly understood. Overall, our study provides the molecular mechanisms underpinning the increased accumulation of fatty acids under nitrogen deprivation conditions.

## Figures and Tables

**Table 1:** Results of qPCR validation of RNA-seq experiments.

Gene ID	KEGG Pathway Annotation	log_2_FC (qPCR)	log_2_FC (RNA-Seq)	Regulation
SW8_14063	Polyunsaturated fatty acid synthase gene *pfa*B	2.98	2.82	up
SW8_00409	S-(hydroxymethyl) glutathione dehydrogenase/alcohol dehydrogenase [EC:1.1.1.284/1.1.1.1]	1.47	1.24	up
SW8_03618	Mitochondrial trans-2-enoyl-CoA reductase [EC:1.3.1.38]	2.01	1.50	up
SW8_10781	Acyl-CoA oxidase [EC:1.3.3.6]	2.21	2.55	up
SW8_11882	[acyl-carrier-protein] S-malonyl transferase [EC:2.3.1.39]	0.32	3.14	up
SW8_13715	Pyruvate dehydrogenase E2 component (dihydrolipoamide acetyltransferase) [EC:2.3.1.12]	1.66	1.08	up
SW8_10069	Dual specificity phosphatase 10 [EC:3.1.3.16/3.1.3.48]	1.72	7.05	up
SW8_10649	Oxidoreductase NAD-binding domain; Ferric reductase NAD binding domain	1.83	3.22	up
SW8_08004	Long-chain acyl-CoA synthetase [EC:6.2.1.3]	−1.70	−1.57	down
SW8_09042	Long-chain-fatty-acid--CoA ligase ACSBG [EC:6.2.1.3]	−1.64	−1.30	down
SW8_11448	Palmitoyl-protein thioesterase [EC:3.1.2.22]	−1.29	−1.20	down
SW8_12262	Enoyl-CoA hydratase [EC:4.2.1.17]	−1.90	−1.99	down

The strain *Aurantiochytrium* sp. PKU#SW8 was used in RNA-seq experiments; The strain was cultivated on glucose (20 g/L) as the carbon source and MSG (0 g/L and 1.2 g/L) as the nitrogen source for 96 h.

## Data Availability

The raw sequences have been deposited in NCBI (National Center for Biotechnology Information) under the BioProject PRJNA863927.

## Supplementary Materials

The following supporting information can be downloaded at: https://www.mdpi.com/article/10.3390/md20100621/s1, Figure S1: Cluster of Orthologous Groups (COG) function classification of unigenes. The horizontal coordinates are function classes of COG, and the vertical coordinates are numbers of unigenes in one class. The notation on the right is the full name of the function class on the X-axis.; Figure S2: cluster of KEGG pathway function classification of unigenes. The vertical coordinate represents the names of the KEGG metabolic pathway and the horizontal coordinate is the number of genes annotated to the pathway. The values in percentage indicate the proportion of the genes annotated to the pathway.; Figure S3: KEGG pathway enrichment of the differentially expressed genes.; Table S1: primers used in qPCR experiments for the RNA-seq data validation.; Table S2: list of new genes discovered through RNA-seq data analysis.; Table S3: list of genes with functional annotations.
