# Supplementary material for: Nitrogen Starvation Enhances the Production of Saturated and Unsaturated Fatty Acids in Aurantiochytrium sp. PKU#SW8 by Regulating Key Biosynthetic Genes
Source: Mar Drugs. 2022 Sep 30;20(10):621. doi: 10.3390/md20100621 (PMC9605394; doi:10.3390/md20100621)
Supplement: Supplementary file 1 [file marinedrugs-20-00621-s001.zip › marinedrugs-1902343-supplementary.pdf]

# Supplementary Information

# Nitrogen Starvation Enhances the Production of Saturated and Unsaturated Fatty Acids in Aurantiochytrium sp. PKU#SW8 by Regulating Key Biosynthetic Genes

**Xiaohong Chen [1,2,†], Yaodong He [1,3,†], Lu Liu [1], Xingyu Zhu [1], Biswarup Sen [1,*] and Guangyi Wang [1,4,5,*]**

[1] Center of Marine Environmental Ecology, School of Environmental Science and Engineering, Tianjin University, Tianjin 300072, China

[2] Marine Traditional Chinese Medicine Research Center, Qingdao Academy of Chinese Medical Sciences, Shandong University of Traditional Chinese Medicine, Qingdao 266114, China
[3] School of Fishery, Zhejiang Ocean University, Zhoushan 316022, China;
[4] Frontiers Science Center for Synthetic Biology, Key Laboratory of Systems Bioengineering (MOE), Tianjin University, Tianjin 300072, China
[5] Center for Biosafety Research and Strategy, Tianjin University, Tianjin 300072, China
* Correspondence: bsen@tju.edu.cn (B.S.); gywang@tju.edu.cn (G.W.)
† The two authors contributed equally to this paper.

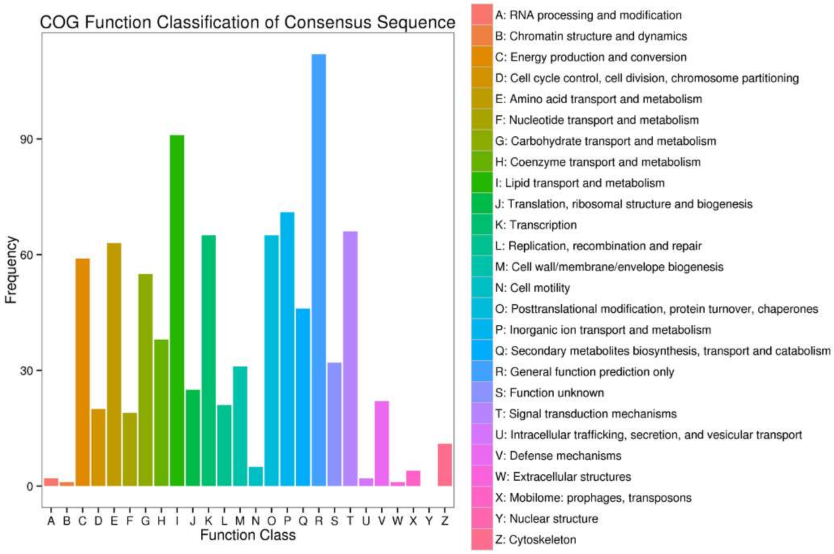


**Figure S1** Cluster of Orthologous Groups (COG) function classification of unigenes. The horizontal coordinates are function classes of COG, and the vertical coordinates are numbers of unigenes in one class. The notation on the right is the full name of the function class on the X-axis.

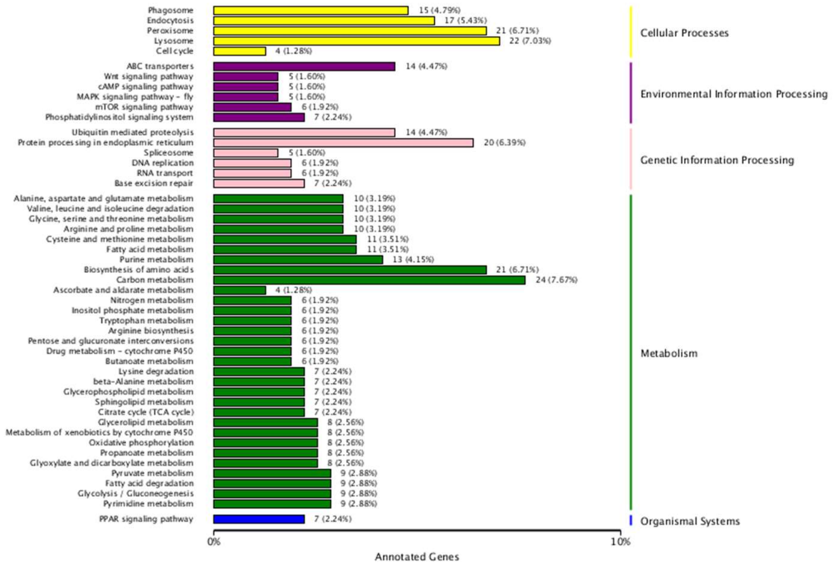


**Figure S2** Cluster of KEGG pathway function classification of unigenes. The vertical coordinate represents the names of the KEGG metabolic pathway and the horizontal coordinate is the number of genes annotated to the pathway. The values in percentage indicate the proportion of the genes annotated to the pathway.

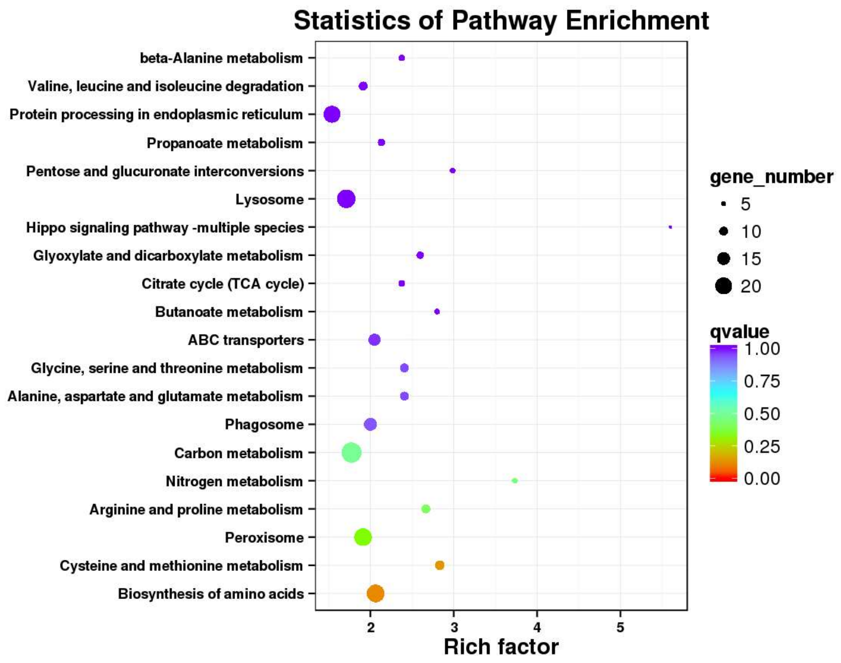


**Figure S3** KEGG pathway enrichment of the differentially expressed genes.

**Table S1** Primers used in qPCR experiments for the RNA-seq data validation.

| Names | Primers (5-3′) |
|---|---|
| 14063-F | ACCCAAGCCACTGTCAAG |
| 14063-R | GTTGGTCTTGGCGTTTAC |
| 00409-F | CGACAACAACAAGAAGGAG |
| 00409-R | CGGTCATCTTAACGAGAAC |
| 03618-F | TGAACTACGGCGATGAAC |
| 03618-R | CGAGGCTAGAGCACATAC |
| 10781-F | GGAGTTACAGGATCTTATTCG |
| 10781-R | TTCATAGACGCCAAGGTAG |
| 11882-F | GCCTTACAAGAGTGGAACT |
| 11882-R | GCAATTCTATCGCTTCAAGT |
| 13715-F | ATGGAGAGTGGTGGCATT |
| 13715-R | GAGGATCTTGGCGAGGAA |
| 10069-F | CGCAATCCTCTACCTCAT |
| 10069-R | CTGTCACGATTCTTCTCAAG |
| 10649-F | GAATGCTATAACGCCAAGAG |
| 10649-R | CGGTTCTGTCTGAATGAGA |
| 08004-F | CTCCGTATCATTGACCGTAA |
| 08004-R | GCAACAAGGCAACTCTGA |
| 09042-F | GTCCTTCCTGGCTCTGAA |
| 09042-R | ACCTTGCTCTTCTTCTGTAG |
| 11448-F | CCTGAAGATACTACGAATGGT |
| 11448-R | ACGGCAGAGAATCCTTGT |
| 12262-F | GAAGGAGGCTGTCAATGTA |
| 12262-R | GACGAAGGCAGTCATACC |
| Actin gene-F | ATGGCGGTAGTCTTCAACAC |
| Actin gene-R | TTTCATTTAGCTCCTTCTGC |
